# Occupational exposure to Baijiu fermentation environments alters nasal microbiota composition: a comparative analysis of Strong-flavor and Sesame-flavor production systems

**DOI:** 10.1128/spectrum.02202-25

**Published:** 2026-08-13

**Authors:** Xiangyang Ge, Jiaojiao Song, Longyun Zhang, Congzhi Zhang, Yong Yang, Xiaoyong Kong, Fengxue Guo, Chun Pu

**Affiliations:** 1 School of Biological and Materials Engineering, Suqian University 164427, , Suqian, Jiangsu, China; 2 Yanghe Distillery Co., Ltd, Suqian, Jiangsu, China; 3 School of Biotechnology, Jiangnan University 546332, Wuxi, Jiangsu, China; University of Mississippi, University, Mississippi, USA

**Keywords:** Baijiu fermentation, nasal microbiota, microbial diversity, source tracking, occupational exposure

## Abstract

Traditional Chinese Baijiu fermentation environments harbor complex microbial consortia critical for product quality, yet their effects on human nasal microbiota remain poorly characterized. This cross-sectional study compared nasal microbiota profiles of Strong-flavor (*n* = 25) and Sesame-flavor (*n* = 22) Baijiu workers against non-exposed individuals (*n* = 30) using 16S rRNA sequencing of environmental samples and nasal swabs. After adjusting for age and working age, no significant differences in Shannon diversity or species richness were observed among the three groups (ANCOVA, *P* > 0.05). However, community structure differed significantly (PERMANOVA, marginal *R*² = 0.075, *P* = 0.001), with Strong-flavor workers clustering closer to non-exposed individuals than Sesame-flavor workers. FEAST-based source tracking identified entry-pit fermented grains, Daqu, and air as primary contributors, accounting for 18.63% and 21.39% of nasal microbiota in Strong- and Sesame-flavor workers, respectively. ALDEx2 revealed six genera depleted in both exposed groups (FDR < 0.05): *Caproiciproducens*, *Petrimonas*, *Proteiniphilum*, *Sporosarcina*, *Syntrophomonas,* and an uncultured genus. No genera were significantly enriched in either exposed group. These findings indicate occupational exposure to Baijiu fermentation environments reduces the nasal carriage of several anaerobic taxa without altering overall alpha diversity.

**IMPORTANCE:**

This study is the first to systematically compare how two major Baijiu fermentation environments—Strong-flavor and Sesame-flavor—affect the nasal microbiota of workers. By integrating environmental and nasal microbiome data, we show that entry pit grains, Daqu, and air are the main known sources of bacteria found in workers’ noses. Occupational exposure in both production systems depletes several anaerobic genera, while overall microbial diversity remains unchanged. These findings connect microbial ecology with occupational health and support practical interventions: improving Daqu storage and ventilation, routine microbial surveillance, and preserving traditional fermentation practices without compromising safety. This work advances our understanding of human environment microbial exchange in traditional food systems with implications for global fermentation industries.

## INTRODUCTION

Baijiu, a traditional Chinese distilled spirit with profound cultural and historical significance, is produced through complex fermentation processes driven by environmental and microbial factors (1–3). The unique aroma and flavor profiles of different types of Baijiu, such as Strong-flavor and Sesame-flavor Baijiu, are largely determined by the specific microbial consortia involved in the fermentation process (4–6). Strong-flavor and Sesame-flavor are two traditional and well-established categories of Chinese Baijiu, defined by their distinct production processes and aroma profiles (1, 6). Understanding the impact of these microbial communities on both the product and the individuals involved in its production is crucial for optimizing production processes and ensuring the health and safety of workers.

Recent studies have underscored the importance of microbial diversity in traditional fermentation processes, particularly in shaping the quality, flavor, and safety of fermented products. The microbial composition of the fermentation environment has been shown to significantly influence the characteristics of the final product. Specifically, Jin et al. demonstrated that the microbial communities inhabiting Baijiu fermentation pit are highly diverse and play a pivotal role in determining the flavor profiles of Baijiu (1). Similarly, Hao et al. identified key microbial taxa associated with the production of volatile compounds in Baijiu, emphasizing the role of microbial interactions in flavor development (3).

Quantitative microbial source tracking has further elucidated the contribution patterns of environmental microorganisms during Baijiu fermentation. Using SourceTracker, a previous study (7) assessed the contributions of various environmental microbiota to the fermentation process. The results indicated that Daqu contributed 9.10%–27.39% of the bacterial communities and 61.06%–80.00% of the fungal communities. In contrast, the surrounding production environment—including outdoor ground, indoor ground, tools, and other unidentified sources—contributed 62.61%–90.90% of the bacterial communities and 20.00%–38.94% of the fungal communities. Collectively, these findings demonstrate that the production environment serves as the predominant bacterial reservoir in the fermentation system, implying that workers are continuously exposed to a substantial load of environmental bacteria within the fermentation workshop.

The nasal microbiome is an important component part of the human microbiome and is closely associated with human health and disease (8, 9). Due to nasal exposure to the external environment, nasal microbiomes are regarded as more dynamic than those found in the gut (10). Similar microbial exchange between occupational environments and human microbiomes has been observed in other settings; for example, bakers’ hands harbor a greater diversity of sourdough-associated microbes than the average population (11). Consequently, the nasal microorganisms of individuals working in these environments are more vulnerable to the influence of microbial communities in the air and other environmental factors (12). Notably, fungi are rarely stably colonized in the human nasal cavity with extremely low abundance and detection rate (13), and most airborne fungi only exist as transient environmental spores rather than resident microbiota (14); therefore, most current human nasal microbiome studies predominantly focus on bacterial communities via 16S rRNA sequencing, while neglecting fungal components (12, 15–17).

In the context of Baijiu production, the interaction between environmental microbiota and human microbiota remains poorly understood. The current study aims to investigate the impact of different Baijiu production environments on the nasal microbiome of individuals involved in the production process. Specifically, we focus on Strong-flavor and Sesame-flavor Baijiu production environments, examining how these environments influence the microbial diversity and composition in the nasal cavities of workers. By comparing the nasal microbiomes of individuals exposed to different production environments with those of non-exposed individuals, we aim to determine whether occupational exposure alters microbial diversity and community composition and to identify unique microbial signatures associated with each type of Baijiu production, after controlling for potential confounders (age and working age). This study aims to investigate the impact of different Chinese Baijiu fermentation environments on the nasal microbiota of workers. We sampled workers from fermentation workshops of Strong-flavor and Sesame-flavor Baijiu and analyzed the structure of their nasal microbial communities. Additionally, we collected samples from the corresponding fermentation environments, including air, pit-fermented grains, and Medium/High-temperature Daqu, to evaluate the influence of these environmental factors on the nasal microbiota of workers. Through this study, we aim to elucidate the mechanisms by which Baijiu fermentation environments affect the nasal microbiota of workers, providing a scientific basis for optimizing Baijiu fermentation environments and protecting worker health. By elucidating the microbial interactions between Baijiu production environments and human nasal microbiomes, this study contributes to the broader understanding of microbial ecology in traditional fermentation processes. The findings have implications for improving production practices, ensuring worker health, and preserving the unique characteristics of traditional Baijiu.

## MATERIALS AND METHODS

### Study design and sampling

A cross-sectional study was conducted in three Strong-flavor Baijiu workshops (*n* = 25 workers), three Sesame-flavor Baijiu workshops (*n* = 22 workers), and three non-exposed packaging workshops (*n* = 30 controls) in Jiangsu Province, China. Nasal swabs were collected from the posterior nasal cavity using sterile medical swabs (Medico, China). Environmental samples included air (*n* = 21, collected via Junroy air sampler), entry-pit fermented grains (*n* = 20), exit-pit fermented grains (*n* = 17), pit mud (*n* = 10), Medium/High-temperature Daqu (*n* = 13), High-temperature Daqu (*n* = 6), and Sesame-flavor Qu (*n* = 6). All samples were immediately stored at −80°C until processing.

Non-exposed individuals were recruited from three packaging workshops located within the same distillery complex as the fermentation workshops. Individuals in packaging workshops perform finished-product packaging tasks in areas physically separated from the active fermentation lines; they do not handle Daqu, fermented grains, or other microbial-rich raw materials. This design allowed us to isolate the effects of direct fermentation-related microbial exposure while controlling for background environmental factors (the same geographic region, similar socio-economic conditions, and comparable dietary habits). Daqu fermentation workshops were not selected as controls because they would introduce substantial independent microbial exposure due to intensive bioaerosol release, thus failing to provide a true baseline for comparison (12, 17).

### DNA extraction and sequencing

The total genomic DNA of the samples was extracted according to the manufacturer’s instructions of the DNeasy PowerSoil Pro Kit (Shanghai, China) and stored at −20°C for subsequent analysis. The quality and integrity of the extracted DNA were assessed using a QubitTM 4 Fluorometer (Shanghai, China) and a Qsep1 Bio-Fragment Analyzer (Taiwan, China), respectively. The bacterial analysis, the 16S rRNA gene in the V3-V4 hypervariable region, was amplified using primers 338F (5′-ACTCCTACGGGAGGCAGCA-3′) and 806R (5′-GGACTACHVGGGTWTCTAAT-3′) (18). Following amplification, PCR products were purified and normalized to suitable concentrations for library preparation using the AMPPure XP Nucleic-acid Purification Kit (USA) and a MiSeq Reagent Kit V3 (600 cycles) (Illumina, USA). Subsequently, the samples were sequenced using the Illumina MiSeq platform with a 2 × 300 bp read length (19). Quality adjustment was performed using Fastp, followed by primer trimming with QIIME2 (2021.2.0). The DADA2 pipeline was utilized for further processing (20). For the classification of bacterial and fungal amplicon sequence variants (ASVs), the SILVA v138 and UNITE databases were employed, respectively.

### Fermentation processes of Strong-flavor and Sesame-flavor Baijiu

Strong-flavor Baijiu (e.g., Lu zhou Lao jiao, Yang he) employs a solid-state fermentation process in mud pits, which are reused for decades to cultivate a stable microbial ecosystem (4, 21). The process includes

Raw material preparation: sorghum (60%–70%), rice (10%–15%), wheat (10%–15%), and corn (5–10%) are crushed and mixed.

Fermentation: the mixture is layered into mud pits (depth: 2–3 m) and inoculated with Medium/High-temperature Daqu (wheat-based Daqu, fermented at 50–60°C for 30–40 days). Fermentation occurs in three phases, initial stage (0–15 days, 25°C–30°C): dominated by *Lactobacillus* and *Saccharomyces*, producing ethanol and organic acids (21). Middle stage (16–30 days, 30°C–40°C): *Bacillus* and *Clostridium* thrive, generating caproic acid and esters (18). Final stage (31–60 days, natural cooling): microbial activity declines and flavor compounds (e.g., ethyl caproate) accumulate (4).

Distillation: fermented grains, raw material, and rice husk are steam-distilled, and the distilled liquid is stored in pottery jars.

The solid residue after distillation is mixed with Medium/High-temperature Daqu, added at 20%–25% of the raw material weight, and subsequently fermented in pits.

Sesame-flavor Baijiu (e.g., Jingzhi) combines Daqu and stacked fermentation to develop its signature roasted sesame aroma (6, 22):

Raw material preparation: sorghum (80%–90%), wheat (10%–20%) are crushed, and mixed.

Stacked fermentation: the mixture is piled into mounds (height: 1–1.5 m) for aerobic fermentation at 50°C–55°C for 24–48 h, enriching thermophiles (e.g., *Bacillus licheniformis*) and facilitating Maillard reactions to produce pyrazines (e.g., tetramethylpyrazine) (22, 23).

Pit fermentation: the stacked grains are transferred to stone pits for anaerobic fermentation (45–60 days, 35°C–40°C), where *Lactobacillus* and *Pediococcus* further degrade substrates (6).

First steam: fermented grains and rice husk are steam-distilled, the resulting distilled liquid is stored in a pottery jar.

Second steam: the solid residues from the first steam are combined with raw materials and subsequently steamed.

Following second steam, three types of Daqu are introduced: High-temperature Daqu, which is wheat-based and fermented at 60°C–65°C for 40–50 days, is added at 25%–30% of the raw material weight; Medium/High-temperature Daqu, which is added at 5%–10% of the raw material weight; and Sesame-flavor Qu, a culture of high-quality fungi and bacteria formed on bran, also added at 5%–10% of the raw material weight. These components are then thoroughly mixed and subjected to stacked fermentation.

### Statistical analysis

All statistical analyses were performed in R (version 4.2.0). Alpha diversity (Shannon index and species richness) was compared among groups using analysis of covariance (ANCOVA) with age and working age as covariates, followed by post-hoc estimation of marginal means using the emmeans package. Beta diversity was assessed using Bray-Curtis distances and visualized by principal coordinates analysis (PCoA). Permutational multivariate analysis of variance (PERMANOVA, adonis2 in vegan) was performed with 999 permutations using the model dist_bray ~ Group + age + working age and by = “margin” to obtain marginal effects. Analysis of similarities (ANOSIM) was performed using the vegan package. Detailed statistical outputs are provided in Table S1. For comparisons involving more than two groups (e.g., different sample types within each flavor system in Fig. 2), alpha diversity was analyzed using Kruskal-Wallis tests followed by Dunn’s post-hoc tests with Benjamini-Hochberg adjustment. Beta diversity differences were assessed by ANOSIM, with R and *P* values shown in the figures.

For differential abundance analysis, we used ALDEx2 (version 1.30.0) (24), which employs a centered log-ratio (clr) transformation and a generalized linear model to account for the compositional nature of microbiome data. Age and working age were included as covariates in the model (~ Group + Age + Working age), with “non-exposed” as the reference level. *P* values were adjusted for multiple testing using the Benjamini-Hochberg (BH) procedure, and genera with an adjusted *P* value (false discovery rate, FDR) <0.05 were considered significantly different between groups.

### Microbial community and source tracking analysis

Genus-level stacked barplots were generated using the microeco package (25), showing the major genera averaged per group (NE, STR, SES). Shared ASVs between sample types were identified using the intersect function in R. For each shared set, relative abundance in the recipient (nasal cavity) was calculated, and bipartite networks were built with ggraph and tidygraph (edge width ∝ shared ASV count; labels show count and nasal relative abundance). Source tracking was performed using FEAST (26) with nasal samples as sinks and all environments as potential sources. Source proportions were averaged per flavor system and displayed as pie charts. Chord diagrams (circlize) illustrated genus-level transfer flows from sources to the nasal cavity (top five genera; ribbon width ∝ abundance).

## RESULTS

### Study population and environmental sample characteristics

Details of the sampling population can be found in Table 1. In total, three Strong-flavor and three Sesame-flavor Baijiu workshops were included, along with NE from three packaging workshops. Nasal swabs were collected from 22 to 30 workers in each group (total *n* = 77), all of whom were male. The mean age of the sampled individuals ranged from 38 to 43 years, and the mean working age varied from 8.7 to 16.3 years. Details of the sampling environmental can be found in Table 2. Two to four air samples were collected at each location (total *n* = 21). Additionally, six to seven samples of M/H Daqu were collected. In this study, two types of Baijiu production utilized distinct Daqu-making workshops to produce M/H Daqu, and to ensure accurate analysis, samples were collected separately (total *n* = 13). Six samples each of H Daqu and S/f Qu were collected. Three to four entry grains samples were collected at each location (total *n* = 20), and two to four exit grains samples were collected at each location (total *n* = 17). Furthermore, two to four pit mud samples were collected at each Strong-flavor Baijiu location (total *n* = 10).

**TABLE 1:** Characteristics of the study population

Location	No. of sampled individuals	No. of sampled male individuals	Age, mean (range) (years)	Working age, mean (range) (years)
Strong-flavor Baijiu				
1	10	10	40.9 (35–43)	14.7 (10–18)
2	8	8	39.6 (34–46)	16.3 (12–26)
3	7	7	42.6 (34–52)	11.9 (9–13)
Sesame-flavor Baijiu				
4	9	9	38.3 (28–55)	8.7 (2–14)
6	6	6	39.7 (35–50)	13.3 (12–14)
7	7	7	41 (30–50)	9.1 (5–13)
Non-exposed				
8–10	30	30	39.4 (31–54)	14.7 (2–34)

**TABLE 2:** Characteristics of the study environment

Location	No. of sampled exit grains	No. of sampled entry grains	No. of sampled pit mud	No. of air samples	No. of sampled M/H Daqu	No. of sampled H Daqu	No. of sampled S/f Qu
Strong-flavor Baijiu							
1	2	4	4	3	7		
2	3	3	3	2			
3	3	3	3	4			
Sesame-flavor Baijiu							
4	2	3		4	6	6	6
6	3	3		4			
7	4	4		4			

### Baijiu production environments influence the nasal microbiome of individuals

**Fig 1:**
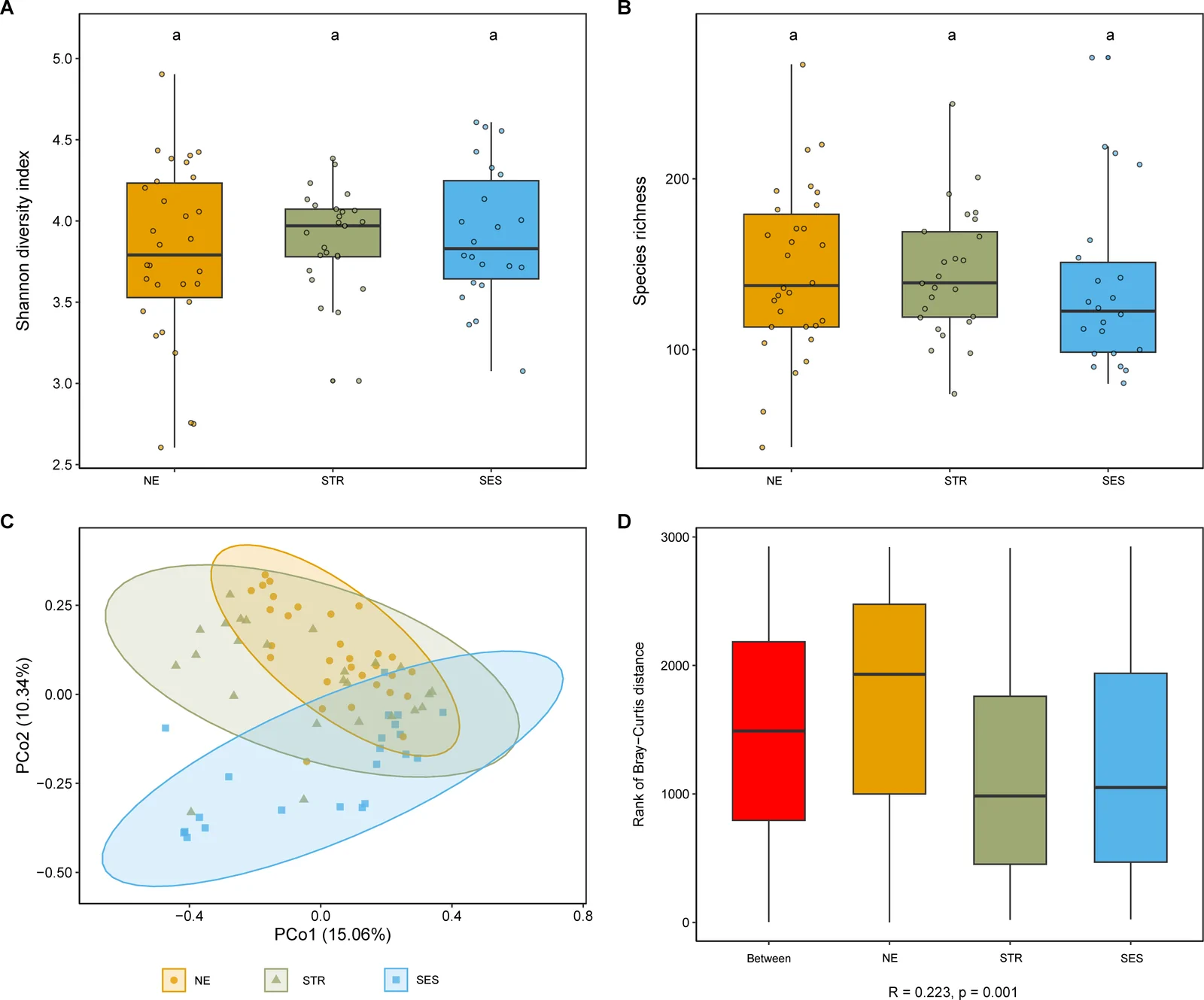
Alpha- and beta-diversity analyses of nasal samples from NE, STR, and SES. (**A, B**) Estimated marginal means (±95% CI) for Shannon diversity index and species richness after adjusting for age and working age by ANCOVA. No significant differences were observed (Shannon: *P* = 0.611; Species: *P* = 0.733). (**C**) Principal coordinates analysis (PCoA) plot based on Bray–Curtis distances, PERMANOVA with age and working age as covariates confirmed a significant group effect (marginal *R*² = 0.075, *P* = 0.001; Table S2). (**D**) Boxplot of rank distances from ANOSIM (*R* = 0.223, *P* = 0.001). NE, non‑exposed individuals; STR, Strong‑flavor Baijiu workers; SES, Sesame‑flavor Baijiu workers; entry grains, entry‑pit fermented grains; exit grains, exit‑pit fermented grains; M/H Daqu, medium/high‑temperature Daqu; H Daqu, high‑temperature Daqu; S/f Qu, Sesame‑flavor Qu.

All 5,915 amplicon sequence variants (ASVs) were classified into 25 phyla and 260 families. The phyla Firmicutes, Proteobacteria, Actinobacteria, and Bacteroidetes accounted for the majority of ASVs, with a mean relative abundance of at least 98.90% across all sample groups. After adjusting for age and working age using analysis of covariance (ANCOVA), no significant differences in Shannon diversity index (*P* = 0.611) or species richness (*P* = 0.733) were observed among the three groups (Fig. 1A and B; Table S1.). The estimated marginal means (with 95% confidence intervals) for Shannon index were 3.77 (3.60–3.93) for NE, 3.90 (3.72–4.07) for STR, and 3.95 (3.75–4.15) for SES. For species richness, the adjusted means were 144 (127–161), 145 (126–163), and 139 (119–160), respectively. In contrast, beta diversity analysis using PERMANOVA with age and working age as covariates revealed a significant effect of occupational group on nasal microbial community structure (*R*² = 0.075, *F* = 3.02, *P* = 0.001; Table S2). PCoA plot showed that STR clustered more closely with NE, while SES formed a distinct cluster (Fig. 1C). ANOSIM corroborated these differences (*R* = 0.223, *P* = 0.001; Fig. 1D).

### Different type Baijiu fermentation factors influence the nasal microbiome of individuals

We selected various factors from different Baijiu production environments to analyze their impact on human nasal microecology in the context of Strong-flavor Baijiu and Sesame-flavor Baijiu. For Strong-flavor Baijiu, we selected air, exit grains, entry grains, pit mud, and M/H Daqu. In contrast, for Sesame-flavor Baijiu, we selected air, exit grains, entry grains, M/H Daqu, H Daqu, and S/f Qu.

**Fig 2:**
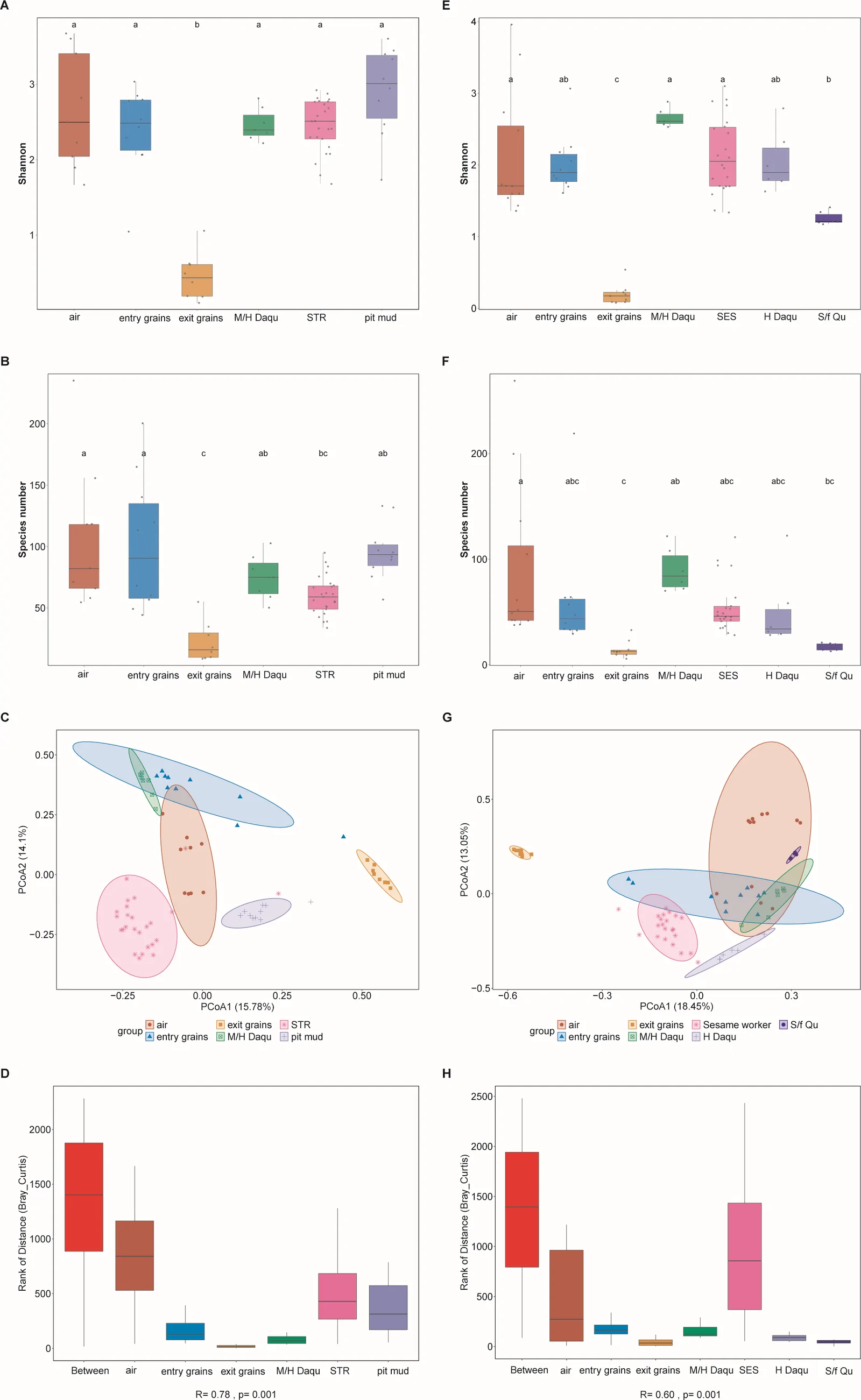
Alpha- and beta-diversity analyses of nasal samples and fermentation environment. (**A, E**) Shannon diversity indices; (**B, F**) species richness. (**C, G**) Principal coordinates analysis (PCoA) plots based on Bray–Curtis distances. (**D, H**) ANOSIM boxplots of beta distances (*R* > 0 indicates greater between-group differences). Different letters indicate significant differences within panels A, B, E, F (*P* < 0.05) determined by Kruskal-Wallis tests with Dunn’s post-hoc correction. ANOSIM confirmed significant differences (D: *R* = 0.78, *P* = 0.001; H: *R* = 0.6, *P* = 0.001). NE, non‑exposed individuals; STR, Strong‑flavor Baijiu workers; SES, Sesame‑flavor Baijiu workers; entry grains, entry‑pit fermented grains; exit grains, exit‑pit fermented grains; M/H Daqu, medium/high‑temperature Daqu; H Daqu, high‑temperature Daqu; S/f Qu, Sesame‑flavor Qu.

In the production of Strong-flavor Baijiu, the exit grains exhibited the lowest Shannon diversity index and species richness (*P* < 0.05, Fig. 2A and B). In contrast, air and entry grains showed higher species richness than STR nasal samples. The weighted PCoA plot (Fig. 2C) revealed distinct clustering patterns between STR and the environmental factors. The microbial profiles of STR were more similar to those of air than to other environmental sources. ANOSIM further confirmed the differences between STR and other factors (*R* = 0.78, *P* = 0.001, Fig. 2D), indicating that inter-group differences were greater than intra-group differences. STR exhibited moderate beta-diversity dispersion compared to other factors.

In the Sesame-flavor system, SES, together with air, entry grains, M/H Daqu, and H Daqu, exhibited the highest Shannon diversity indices. Significant differences were observed between SES and both S/f Qu and exit grains, with exit grains showing the lowest Shannon diversity (*P* < 0.05, Fig. 2E). SES displayed comparable species richness to the other factors (Fig. 2F), whereas exit grains had the lowest species richness among all factors (excluding S/f Qu). The weighted PCoA plot (Fig. 2G) illustrated distinct clustering patterns of SES relative to other factors. The profiles of SES were more similar to those of air, M/H Daqu, H Daqu, and entry grains than to exit grains and S/f Qu. ANOSIM analysis further confirmed significant differences between SES and other factors (*R* = 0.6, *P* = 0.001, Fig. 2H), indicating that inter-group differences exceeded intra-group differences. Additionally, SES appeared to exhibit higher beta-diversity dispersion compared to other factors.

### Overall genus-level composition of nasal microbiota

**Fig 3:**
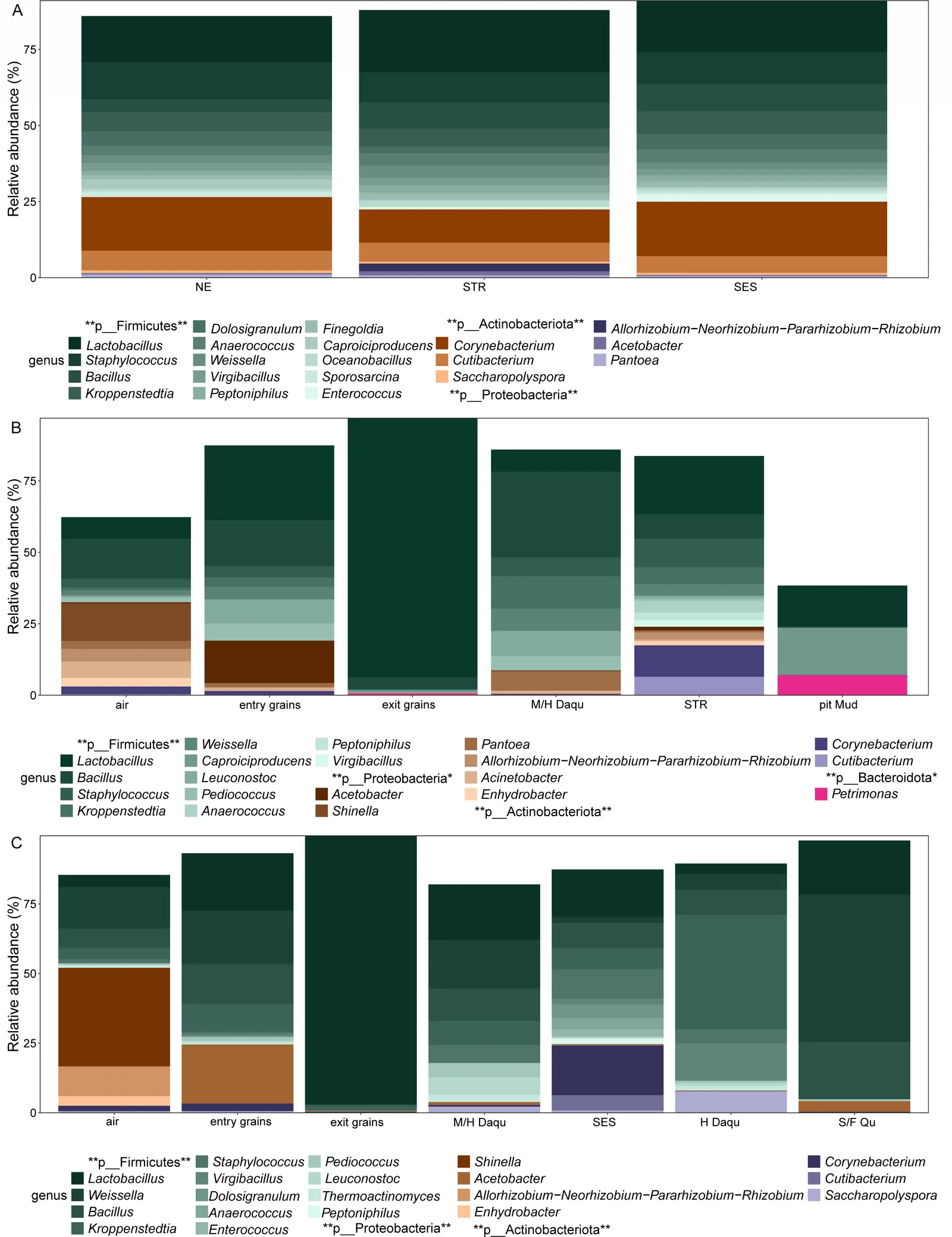
Genus-level relative abundance of nasal and environmental microbiota. Stacked barplots showing mean relative abundance of major genera (>1%). (**A**) Nasal microbiota of NE, STR, and SES. (**B**) Strong-flavor system: environmental samples. (**C**) Sesame-flavor system: environmental samples. Lower-abundance genera are grouped as “Others.” Phylum classification is indicated. NE, non‑exposed individuals; STR, Strong‑flavor Baijiu workers; SES, Sesame‑flavor Baijiu workers; entry grains, entry‑pit fermented grains; exit grains, exit‑pit fermented grains; M/H Daqu, medium/high‑temperature Daqu; H Daqu, high‑temperature Daqu; S/f Qu, Sesame‑flavor Qu.

To assess whether occupational exposure alters the nasal microbial composition, we compared genus-level relative abundances among the three groups (Fig. 3A). In NE, the nasal microbiota was dominated by *Corynebacterium* (17.64%), *Lactobacillus* (15.26%), and *Staphylococcus* (12.09%). STR exhibited a marked increase in *Lactobacillus* (20.41%) and a decrease in *Corynebacterium* (10.90%), while SES showed an intermediate profile (*Lactobacillus* 17.06%, *Corynebacterium* 17.92%). More strikingly, the anaerobic genera *Caproiciproducens* and *Sporosarcina*—abundant in NE (3.12% and 1.76%, respectively)—were drastically reduced in STR (0.13% and 0.18%) and remained low in SES (0.49% and 0.95%). These patterns suggest that occupational exposure selectively depletes oxygen-sensitive anaerobes.

We next characterized the microbial composition of environmental sources in each production system (Fig. 3B and C). In the Strong-flavor system (Fig. 3B), air was dominated by *Bacillus* (13.99%), *Shinella* (13.19%), and *Lactobacillus* (7.59%). Entry grains contained high proportions of *Lactobacillus* (26.16%) and *Bacillus* (16.00%). Exit grains were overwhelmingly dominated by *Lactobacillus* (90.73%). M/H Daqu was enriched in *Bacillus* (30.10%) and *Kroppenstedtia* (11.41%). Pit mud was characterized by *Caproiciproducens* (16.51%), *Lactobacillus* (14.30%), and *Petrimonas* (6.86%). In the Sesame-flavor system (Fig. 3C), air was dominated by *Shinella* (35.33%), *Weissella* (14.98%), and *Allorhizobium-Neorhizobium-Pararhizobium-Rhizobium* (10.62%). Entry grains contained high *Acetobacter* (21.22%), *Lactobacillus* (20.72%), and *Weissella* (19.17%). Exit grains were dominated by *Lactobacillus* (96.58%). M/H Daqu showed abundant *Lactobacillus* (19.92%), *Weissella* (17.50%), and *Bacillus* (11.64%). H Daqu was characterized by *Kroppenstedtia* (41.11%), *Virgibacillus* (13.38%), and *Bacillus* (8.97%). S/f Qu was dominated by *Weissella* (52.97%), *Bacillus* (20.52%), and *Lactobacillus* (19.46%). These distinct environmental profiles provide the source pools for potential microbial transfer to workers’ nasal cavities.

### Differential abundance analysis

**Fig 4:**
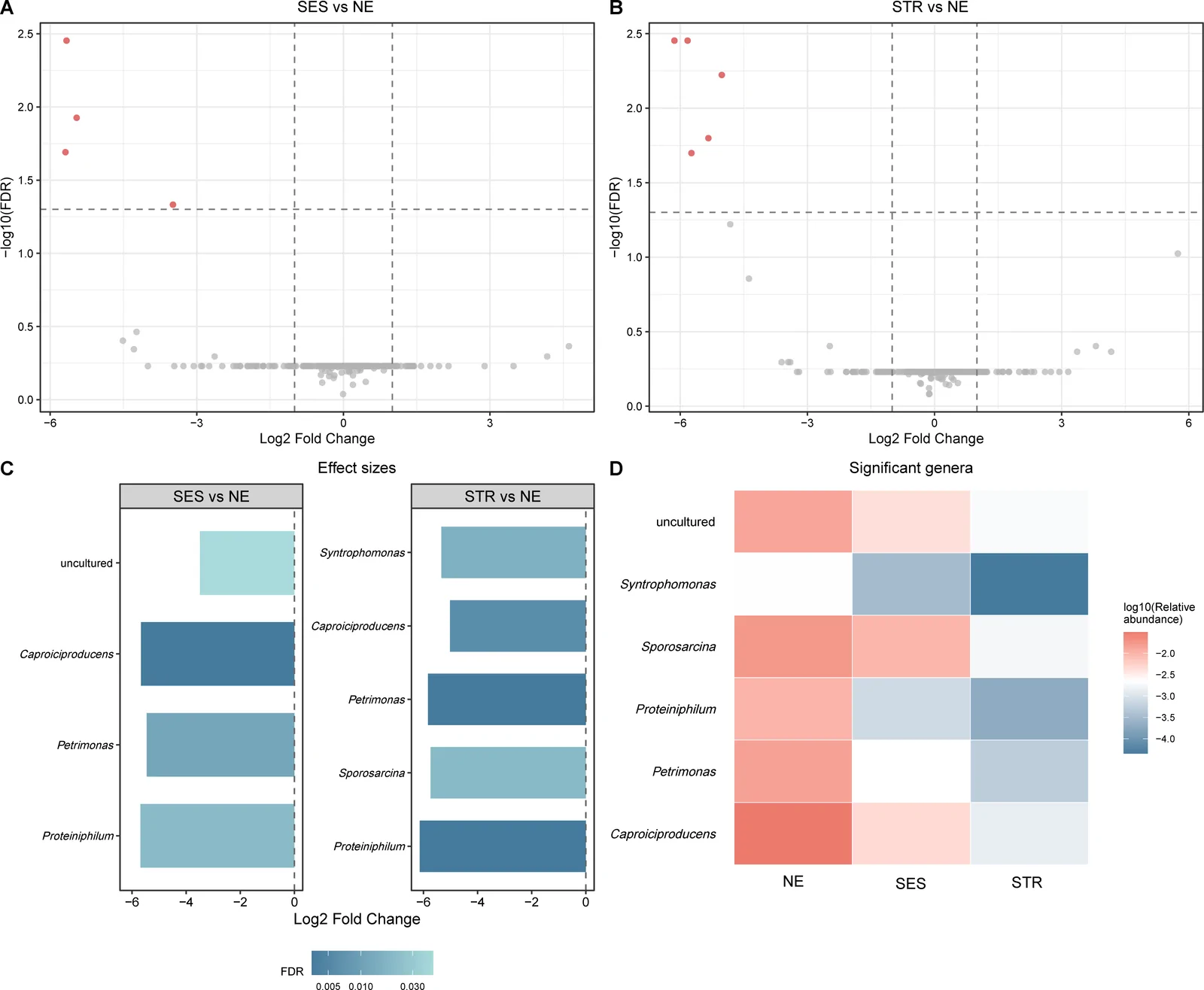
Differential abundance of nasal bacterial genera identified by ALDEx2. (**A**) Volcano plot for SES vs NE. (**B**) Volcano plot for STR vs NE. Red points: FDR < 0.05. Dashed vertical lines: |log₂FC| = 1; dashed horizontal line: FDR = 0.05. (**C**) Barplot of log₂FC for significantly different genera (FDR < 0.05). (**D**) Heatmap of log₁₀-transformed mean relative abundance across the three groups. FDR, false discovery rate; log₂FC, log₂ fold change. NE, non‑exposed individuals; STR, Strong‑flavor Baijiu workers; SES, Sesame‑flavor Baijiu workers; entry grains, entry‑pit fermented grains; exit grains, exit‑pit fermented grains; M/H Daqu, medium/high‑temperature Daqu; H Daqu, high‑temperature Daqu; S/f Qu, Sesame‑flavor Qu.

To identify genera altered by occupational exposure after adjusting for age and working age, we performed ALDEx2. Six genera showed significantly lower abundances in exposed workers compared to NE (FDR < 0.05; Fig. 4). Volcano plots (Fig. 4A and B) display log₂ fold change vs—log₁₀(FDR) for all genera, with red points indicating significant genera. The barplot (Fig. 4C) shows effect sizes for each comparison. Three genera*—Caproiciproducens*, *Petrimonas*, and *Proteiniphilum*—were significantly lower in both STR and SES. An uncultured genus was lower only in SES, while *Sporosarcina* and *Syntrophomonas* were lower only in STR. No genus showed a significantly positive log₂ fold change (i.e., no enrichment in either exposed group). The heatmap (Fig. 4D) visualizes the log₁₀-transformed mean relative abundance of these six genera across the three groups, confirming distinct depletion patterns. The complete ALDEx2 output, including log₂ fold change, raw *P* values, and false discovery rates for all genera, is provided in Table S3.

### Shared and unique ASVs among exposure groups

**Fig 5:**
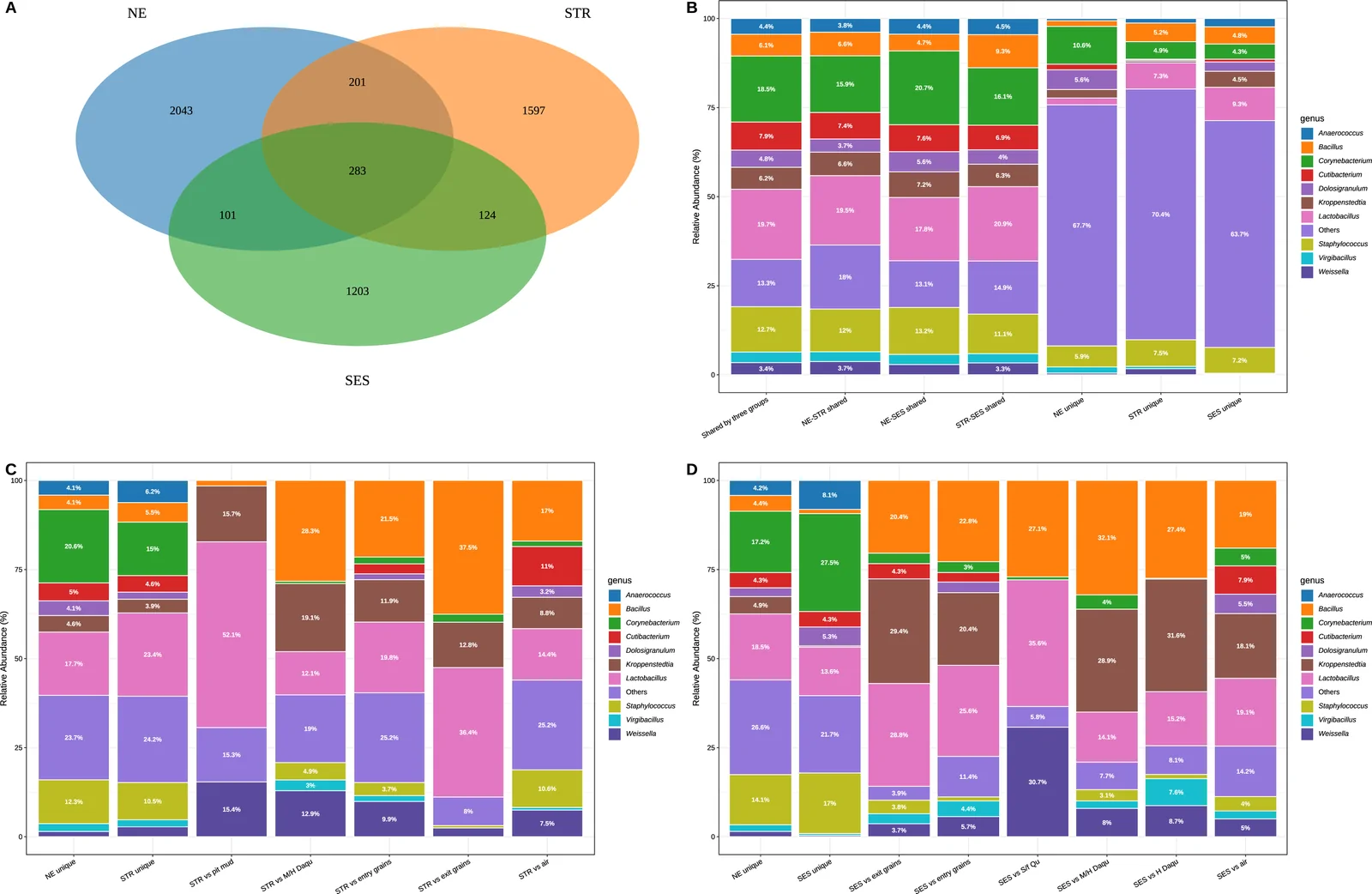
Shared and unique ASV fractions. (**A**) Venn diagram of ASV counts among NE, STR, and SES. (**B**) Genus composition of shared/unique fractions among the three groups. (**C**) Genus composition of unique ASVs (NE_nasal_unique, STR_nasal_unique) and ASVs shared between STR nasal cavity and each environmental source (air, entry grains, exit grains, M/H Daqu, pit mud). (**D**) Genus composition of unique ASVs (NE_nasal_unique, SES_nasal_unique) and ASVs shared between SES nasal cavity and each environmental source (air, entry grains, exit grains, M/H Daqu, H Daqu, S/f Qu). NE, non‑exposed individuals; STR, Strong‑flavor Baijiu workers; SES, Sesame‑flavor Baijiu workers; entry grains, entry‑pit fermented grains; exit grains, exit‑pit fermented grains; M/H Daqu, medium/high‑temperature Daqu; H Daqu, high‑temperature Daqu; S/f Qu, Sesame‑flavor Qu.

We next examined ASV sharing to determine the extent of a core nasal microbiome. A total of 283 ASVs were shared by all three groups, representing the majority of relative abundance in each group (NE: 71.61%, STR: 76.37%, SES: 75.15%; Table S4), indicating a stable core microbiota. NE harbored the highest number of unique ASVs (2,043; 14.68% relative abundance), while STR and SES had fewer unique ASVs (1,597 and 1,203; 10.74% and 9.90%, respectively). The three-way shared fraction (Fig. 5B) was dominated by *Lactobacillus* (19.67%), *Corynebacterium* (18.52%), *Staphylococcus* (12.74%), and *Bacillus* (6.11%). Unique ASVs in NE were enriched in *Corynebacterium* (10.62%) and *Staphylococcus* (5.91%), whereas those in STR and SES showed higher proportions of *Lactobacillus* (7.34% and 9.34%, respectively) and *Bacillus* (5.22% and 4.84%, respectively), mirroring the compositional shifts observed in Fig. 3A.

### Shared and unique ASVs between nasal cavity and environmental sources

To identify which environmental compartments share ASVs with the nasal microbiota, we analyzed ASVs present in workers’ nasal samples relative to each environmental source. Nasal-unique ASVs were defined as those found in a group’s nasal samples but absent from all environmental samples of that system, representing the intrinsic nasal microbiota.

In the Strong-flavor system (Fig. 5C), the nasal-unique ASVs of NE (NE_nasal_unique) were dominated by *Corynebacterium* and *Lactobacillus*, whereas those of STR (STR_nasal_unique) shifted toward *Lactobacillus* (23.4%) with reduced *Corynebacterium* (15.0%). Among the shared fractions, ASVs from air, entry grains, and Daqu contained high proportions of *Bacillus*, *Lactobacillus*, and *Kroppenstedtia*. For example, *Bacillus* comprised 17.0% of air-shared ASVs, 21.5% of entry-grain shared ASVs, and 28.3% of M/H Daqu-shared ASVs. These genera were also abundant in the overall nasal microbiota of STR (Fig. 3A).

In the Sesame-flavor system (Fig. 5D), a similar pattern was observed: air, entry grains, and H Daqu shared ASVs were also dominated by *Bacillus*, *Lactobacillus*, and *Kroppenstedtia*. Notably, *Kroppenstedtia* reached 31.6% in H Daqu-shared ASVs, and *Weissella* was abundant in S/f Qu-shared ASVs (30.7%). These genera were likewise prominent in the nasal microbiota of SES (Fig. 3A). The nasal-unique ASVs of SES (SES_nasal_unique) were enriched in *Corynebacterium* (27.5%), contrasting with the *Lactobacillus*-dominant profile of STR_nasal_unique.

### Quantitative source tracking of nasal microbiota

**Fig 6:**
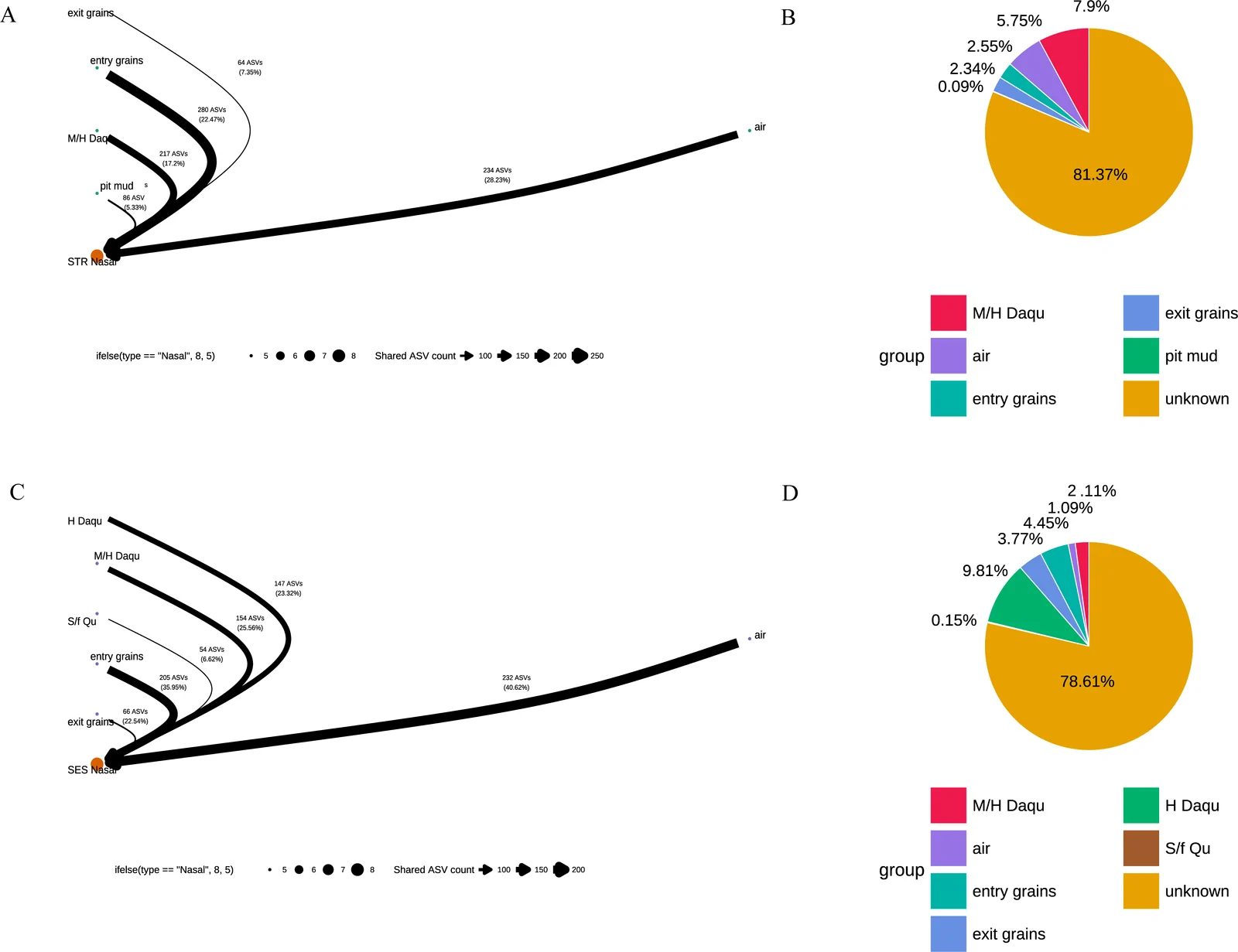
Source tracking of nasal microbiota. (**A**) Bipartite network for STR: edge width ∝ shared ASV count; labels show “ASV count (nasal relative abundance, %).” Top donors: air, entry grains, M/H Daqu. (**B**) FEAST pie chart for STR. (**C**) Bipartite network for SES. Top donors: air, entry grains, H Daqu, M/H Daqu. (**D**) FEAST pie chart for SES. NE, non‑exposed individuals; STR, Strong‑flavor Baijiu workers; SES, Sesame‑flavor Baijiu workers; entry grains, entry‑pit fermented grains; exit grains, exit‑pit fermented grains; M/H Daqu, medium/high‑temperature Daqu; H Daqu, high‑temperature Daqu; S/f Qu, Sesame‑flavor Qu.

To trace the origin of nasal microbiota at the ASV level, bipartite networks (Fig. 6A and C) were constructed to display the number and nasal relative abundance of ASVs shared between each environmental source and workers’ nasal cavities. Air shared the highest number of ASVs with the nasal cavity in both production systems (STR: 234 ASVs, 28.23% nasal relative abundance; SES: 232 ASVs, 40.62%). Entry grains were also major donors (STR: 280 ASVs, 22.47%; SES: 205 ASVs, 35.95%), followed by M/H Daqu in STR (217 ASVs, 17.20%) and, in SES, M/H Daqu (154 ASVs, 25.56%) and H Daqu (147 ASVs, 23.32%). Pit mud contributed modestly in STR (86 ASVs, 5.33%).

To further quantify the proportional contribution of each environmental source, we performed FEAST source tracking (Fig. 6B and D). Known environmental sources explained only 18.63% of the nasal microbiota in STR (Medium/High Daqu: 7.90%; air: 5.75%; entry grains: 2.55%; exit grains: 2.34%; pit mud: 0.09%) and 21.39% in SES (High Daqu: 9.81%; entry grains: 4.45%; exit grains: 3.77%; Medium/High Daqu: 2.11%; air: 1.09%; Sesame Qu: 0.15%). The majority of nasal bacteria remained attributed to unknown sources (81.37% and 78.61%, respectively), underscoring the dominance of the host-adapted core microbiome. Taken together, these quantitative results demonstrate that air, entry grains, and various types of Daqu are the primary identified environmental sources of nasal microbiota despite their modest overall contribution.

Chord diagrams (Fig. S1) visually confirmed that *Bacillus*, *Lactobacillus*, and *Kroppenstedtia* are the dominant genera transferred from environmental sources to the nasal cavity, with air, entry grains, and Daqu (both Medium/High and High-temperature) all serving as important reservoirs for these taxa.

## DISCUSSION

This cross-sectional study investigated the effects of various Baijiu production environments on the nasal microbiome of workers, yielding a series of valuable results. These findings are interconnected with previous research and offer new insights into this field.

### Alpha and beta diversity responses to occupational exposure

After rigorous adjustment for age and working age, we observed no significant differences in alpha diversity among the three groups (Fig. 1A and B; Table S1). This suggests that the core nasal microbiota maintains a stable level of richness and evenness despite prolonged occupational exposure to diverse Baijiu fermentation environments. However, beta diversity analysis revealed a clear separation in community composition (Fig. 1C and D; PERMANOVA, marginal *R*² = 0.075, *P* = 0.001; Table S2), indicating that while overall diversity is preserved, the relative abundances of specific taxa are reshaped by the working environment. This pattern aligns with the “Anna Karenina principle” (AKP) of microbiome stability, which posits that under stress, community composition becomes more variable and individualized among hosts while overall alpha diversity may remain unchanged (27). Consistent with the AKP, our data suggest that occupational exposure to Baijiu fermentation environments—particularly the high-temperature stacking process in Sesame-flavor production—drives a stochastic shift in nasal microbial community assembly without significantly altering species richness or Shannon diversity.

### Depletion of anaerobic taxa and absence of enrichment

The differential abundance analysis (ALDEx2) identified six anaerobic genera that were significantly depleted in exposed workers compared to NE (FDR < 0.05; Fig. 4): *Caproiciproducens*, *Petrimonas*, *Proteiniphilum*, *Sporosarcina*, *Syntrophomonas,* and an uncultured genus. Notably, no genus showed significant enrichment in either exposed group. These genera are typically found in oxygen-limited environments such as animal gastrointestinal tracts, anaerobic digesters, or sediments, where they participate in the degradation of complex organic matter and the production of short-chain fatty acids (28–31). Their strict dependence on reduced conditions makes them particularly vulnerable to oxidative stress (18).

The depletion is unlikely to be caused by the acidic or organic acid-rich conditions of the fermentation environment, as several of these anaerobes have been shown to survive and even thrive in the acidic fermented grains during Strong-flavor Baijiu production (18). Instead, the nasal cavity represents a fundamentally different habitat. Unlike the partially reduced environment of fermented grains, the nasal mucosa is continuously exposed to air, bioaerosols, and high oxygen levels. The workshop environment imposes additional stressors, including elevated ethanol vapor, which has been shown to alter nasal microbiome composition (32). Ethanol can exert direct toxic effects on strict anaerobes by disrupting cell membranes and generating reactive oxygen species (33–35). The combination of oxygen exposure, ethanol vapor, and handling of dry grains creates oxidative conditions that are particularly hostile to oxygen-sensitive anaerobes, which lack key antioxidant enzymes such as superoxide dismutase and catalase (36).

Interestingly, the depletion patterns of these genera differed between the two exposure groups: *Sporosarcina* and *Syntrophomonas* were significantly reduced only in STR, whereas the uncultured genus was reduced only in SES (Fig. 4C). This divergence may reflect subtle differences in the physicochemical profiles of the two fermentation systems—for example, the higher temperature and prolonged stacking in Sesame-flavor production may impose different selective pressures on specific taxa. However, the overall similarity in the depletion of the other four genera across both worker groups suggests a common response to the shared stressors of the Baijiu fermentation environment.

### Environmental sources and the core nasal microbiome

FEAST source tracking revealed that entry grains, Daqu, and air are the primary known environmental sources of nasal microbiota, collectively explaining 18.63% of the nasal microbiota in STR and 21.39% in SES (Fig. 6B and D). The majority of nasal bacteria remained attributed to unknown sources (81.37% and 78.61%, respectively), underscoring the dominance of the host-adapted core microbiome. This high proportion of unknown sources is consistent with previous studies showing that the nasal microbiome exhibits high temporal stability under normal conditions (10). The shared ASV analysis further supported this view: 283 ASVs were shared by all three groups, representing >71% of the relative abundance in each group (Table S4), indicating a stable core nasal microbiota that persists across exposure groups.

The composition of shared ASVs between the nasal cavity and environmental sources (Fig. 5C and D) showed that air, entry grains, and Daqu consistently contributed *Bacillus*, *Lactobacillus*, and *Kroppenstedtia*-genera that were also abundant in the overall nasal microbiota of exposed workers (Fig. 3A). However, the absence of significant enrichment of these genera in exposed workers (Fig. 4) indicates that their proportional increase (e.g., *Lactobacillus* from 15.3% in NE to 20.4% in STR) is driven by the concomitant depletion of other taxa rather than an absolute increase. This “competitive release” phenomenon has been observed in other occupational exposure studies, where environmental perturbation creates vacant niches that are filled by tolerant taxa without a net increase in absolute abundance (12, 37, 38).

### Microbial transfer pathways

Microbial transfer from environment to nasal cavity likely occurs via two pathways: (i) direct contact through skin-to-hand-to-nose interactions during grain handling and (ii) inhalation of bioaerosols generated during open-air operations. The handling of Daqu and fermented grains generates bioaerosols (1–5  μm particles) that remain suspended in workshop air (39). The nasal cavity, with its oxygen-rich mucosa (approximately 32°C), provides an ideal niche for aerobic colonizers, as evidenced by the dominance of *Staphylococcus* and *Corynebacterium* in this habitat (40, 41). This microenvironment promotes oxidative metabolism while suppressing strictly anaerobic taxa, consistent with the depletion patterns we observed (41).

### Limitations

This study has several limitations. First, the cross-sectional design precludes establishing causal relationships; longitudinal studies are needed. Second, while we adjusted for age and working age, other unmeasured confounders (e.g., smoking, diet) may influence the results. Third, due to the very low abundance and detection rates of fungi in the nasal cavity (13, 14), we focused on bacterial communities; future studies should incorporate ITS sequencing to characterize mycobiome transfer. Fourth, we did not quantify absolute microbial loads; future work could employ qPCR to complement relative abundance analysis. Fifth, airborne microbial concentrations were not measured, limiting our ability to directly link exposure dose with nasal microbiota changes.

### Conclusions

In conclusion, occupational exposure to Strong-flavor and Sesame-flavor Baijiu fermentation environments significantly reshapes nasal microbial community composition without altering overall alpha diversity. After adjusting for age and working age, Shannon diversity and species richness remained stable across groups, whereas the community structure of SES diverged markedly from that of NE, with STR showing an intermediate profile. ALDEx2 analysis revealed a consistent reduction of six anaerobic genera in exposed groups, with no genus significantly enriched, indicating that environmental filtering primarily depletes oxygen-sensitive taxa rather than introducing novel colonizers. Source tracking identified entry grains, Daqu, and air as the main known environmental sources although known sources explained only 18%–21% of the nasal community, highlighting the dominance of the host-adapted core microbiome. To mitigate potential dysbiosis and enhance occupational safety, we propose targeted interventions: optimizing Daqu storage conditions to reduce bioaerosol release, improving handling practices for entry grains (e.g., minimizing open-air piling and dust generation), installing advanced ventilation systems, and implementing routine microbial monitoring of workshop air and surfaces. Future research should integrate metagenomic profiling, absolute quantification, and longitudinal health surveillance to further elucidate the mechanistic links between environmental microbes, nasal microbiota, and clinical outcomes.

## Data Availability

Human nasal microbiota sequence data are deposited in the NCBI SRA under BioProject PRJNA1290228. Multi-niche microbiota (fermented grains, pit mud, Qu, air) sequence data are deposited in the NCBI SRA under BioProject PRJNA1290266.

## SUPPLEMENTAL MATERIAL

Supplemental tables and figures spectrum.02202-25-s0001.pdf Tables S1 to S4 and Fig. S1.

ASM does not own the copyrights to Supplemental Material that may be linked to, or accessed through, an article. The authors have granted ASM a non-exclusive, world-wide license to publish the Supplemental Material files. Please contact the corresponding author directly for reuse.
