# Supplementary material for: Occupational exposure to Baijiu fermentation environments alters nasal microbiota composition: a comparative analysis of Strong-flavor and Sesame-flavor production systems
Source: Microbiol Spectr. 2026 Aug 13;14(9):e02202-25. doi: 10.1128/spectrum.02202-25 (PMC13532321; doi:10.1128/spectrum.02202-25)
Supplement: Supplementary file 1 [file spectrum.02202-25-s0001.pdf]

Supplementary Material

Supplementary Table S1. ANCOVA results for alpha diversity indices after adjusting for age and working age.

Group	Diversity index	Estimated marginal mean	SE	95% CI lower	95% CI upper	Group effect p-value
NE	Shannon	3.77	0.0826	3.6	3.93	
STR	Shannon	3.9	0.0887	3.72	4.07	0.611
SES	Shannon	3.95	0.0991	3.75	4.15	
NE	species richness	144	8.67	127	161	
STR	species richness	145	9.32	126	163	0.733
SES	species richness	139	10.4	119	160	

Note: Covariates included in the ANCOVA model were age and working age. The group effect p-value is from the F-test of the Group factor. Post-hoc pairwise comparisons were not significant (Tukey-adjusted $p > 0.05$).

Supplementary Table S2. PERMANOVA results (marginal effects) for beta diversity (Bray–Curtis distance)

Source	Df	SumOfSqs	R^2	F	$Pr(>F)$
Group	2	2.0572	0.07501	3.0216	0.001
age	1	0.3567	0.01301	1.048	0.364
Work age	1	0.228	0.00831	0.6697	0.913
Residual	72	24.5097	0.89365		
Total	76	27.4267	1		

Note: PERMANOVA was performed with 999 permutations using adonis2 with by = "margin". Covariates (age, working age) were included in the model.

Supplementary Table S3. Significant differentially abundant bacterial genera identified by ALDEx2 after adjusting for age and working age (*FDR* < 0.05).

Genus	Comparison	logFC	p value	*FDR*
Caproiciproducens	STR vs NE	-5.0195	2.01×10^{-5}	0.006
Petrimonas	STR vs NE	-5.8262	6.76×10^{-6}	0.0035
Proteiniphilum	STR vs NE	-6.1371	7.65×10^{-6}	0.0035
Sporosarcina	STR vs NE	-5.7356	1.18×10^{-4}	0.02
Syntrophomonas	STR vs NE	-5.3361	8.00×10^{-5}	0.0159
Caproiciproducens	SES vs NE	-5.6656	8.86×10^{-6}	0.0035
Petrimonas	SES vs NE	-5.4585	4.97×10^{-5}	0.0119
Proteiniphilum	SES vs NE	-5.6865	1.37×10^{-4}	0.0204
uncultured	SES vs NE	-3.4877	3.51×10^{-4}	0.0465

Note: Negative logFC indicates lower abundance in the exposed group. FDR was adjusted using the Benjamini-Hochberg method.

Supplementary Table S4. ASV counts and relative abundance of shared and unique regions among NE, STR, and SES

Region	ASV_count	Rel_abund_in_NE (%)	Rel_abund_in_STR (%)	Rel_abund_in_SES (%)
NE unique	2043	14.68	/	/
STR unique	1597	/	10.74	/
SES unique	1203	/	/	9.9
NE-STR shared	201	11.68	8.84	/
NE-SES shared	101	2.04	/	3.4
STR-SES shared	124	/	4.05	11.54
Three-way shared	283	71.61	76.37	75.15

Note: This table lists the precise ASV counts and relative abundances for the Venn diagram in Figure 5A. Relative abundances of unique and shared regions denote the total proportion of corresponding ASVs within each group. "/" stands for non-existent regions .

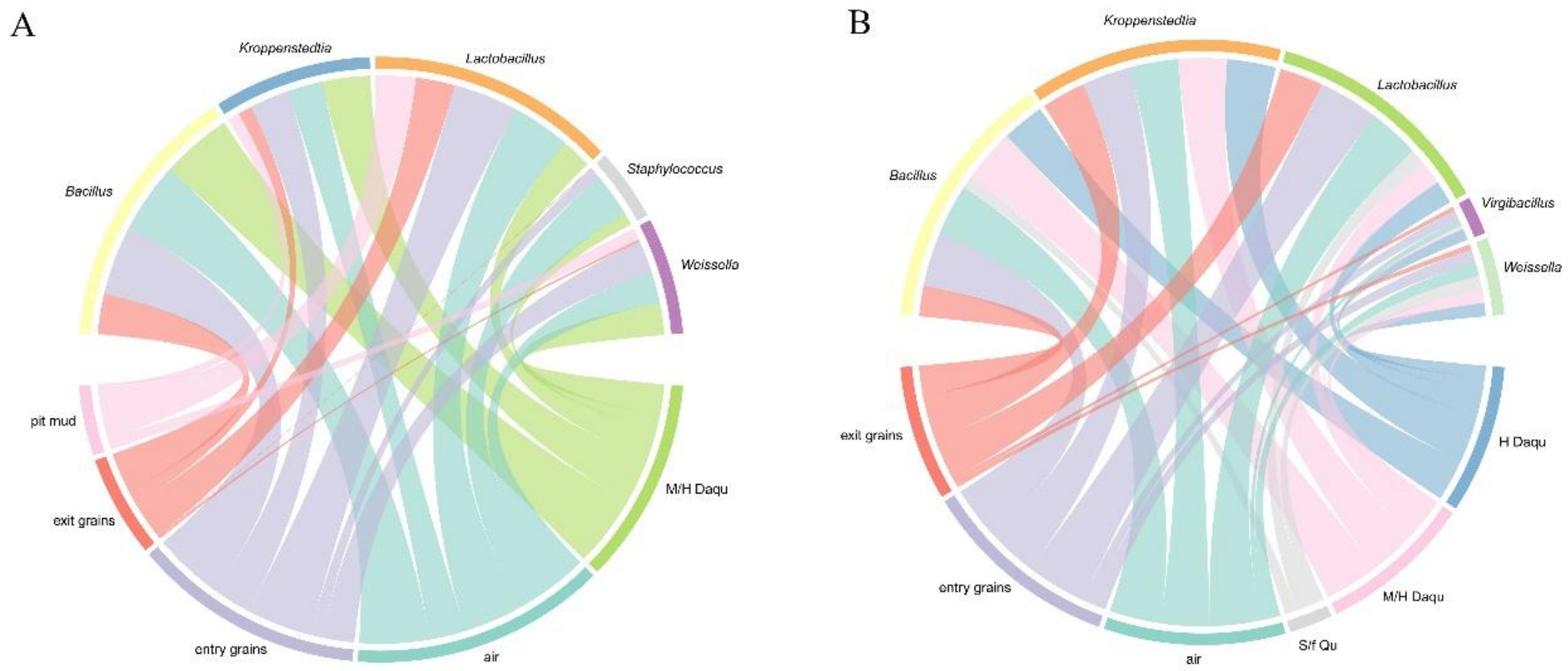

Supplementary Fig S1. Chord diagrams of genus level transfer from environmental sources to the nasal cavity. (A) Strong flavor system. Ribbon width represents the total abundance of a genus contributed by each source. (B) Sesame flavor system. Ribbon width represents the total abundance of a genus contributed by each source.
